# New Insights into VacA Intoxication Mediated through Its Cell Surface Receptors

**DOI:** 10.3390/toxins8050152

**Published:** 2016-05-13

**Authors:** Kinnosuke Yahiro, Toshiya Hirayama, Joel Moss, Masatoshi Noda

**Affiliations:** 1 Department of Molecular Infectiology, Graduate School of Medicine, Chiba University, 1-8-1, Inohana, Chuo-ku, Chiba 260-8670, Japan; noda@faculty.chiba-u.jp; 2 Department of Bacteriology, Institute of Tropical Medicine, Nagasaki University, 1-12-4, Sakamoto, Nagasaki 852-8523, Japan; hirayama@nagasaki-u.ac.jp; 3 Cardiovascular and Pulmonary Branch, NHLBI, NIH, Building 10, Room 6D03, MSC 1590, Bethesda, MD 20892-1590, USA; mossj@nhlbi.nih.gov

**Keywords:** vacuolating cytotoxin (VacA), receptors, receptor-like protein tyrosine phosphatase (RPTP) α, RPTPβ, low-density lipoprotein receptor-related protein-1 (LRP1)

## Abstract

*Helicobacter pylori* (*H. pylori*), a major cause of gastroduodenal diseases, produces VacA, a vacuolating cytotoxin associated with gastric inflammation and ulceration. The *C*-terminal domain of VacA plays a crucial role in receptor recognition on target cells. We have previously identified three proteins (*i.e.*, RPTPα, RPTPβ, and LRP1) that serve as VacA receptors. These receptors contribute to the internalization of VacA into epithelial cells, activate signal transduction pathways, and contribute to cell death and gastric ulceration. In addition, other factors (e.g., CD18, sphingomyelin) have also been identified as cell-surface, VacA-binding proteins. Since we believe that, following interactions with its host cell receptors, VacA participates in events leading to disease, a better understanding of the cellular function of VacA receptors may provide valuable information regarding the mechanisms underlying the pleiotropic actions of VacA and the pathogenesis of *H. pylori*-mediated disease. In this review, we focus on VacA receptors and their role in events leading to cell damage.

## 1. Introduction

*Helicobacter pylori* (*H. pylori*) is a helical-shaped gram-negative microorganism, which colonizes the human stomach and plays important roles in the pathogenesis of not only gut diseases (e.g., gastric inflammation, ulcer, mucosa-associated lymphoid tissue (MALT) lymphoma, gastric cancer [1]), but also chronic idiopathic thrombocytopenic purpura [2]. *H. pylori* produces various virulence factors (e.g., vacuolating cytotoxin (VacA), cytotoxin-associated protein A (CagA), urease, LPS), which are associated with initiating events in the pathogenesis of disease [3,4,5].

VacA secreted from *H. pylori* has a 90-kDa molecular mass. Its C-terminal region of about 55 kDa (p55) plays an important role in binding to toxin receptors on cell membranes [6,7,8,9,10]. The *C*-terminal domain contains two important mid-region subtypes, m1 and m2, which are less than 60% identical in amino acid sequence [11]. These two types of VacA have different affinities for cell-surface receptors [8,12,13] and show different cell specificities [14]. Analysis of p55 crystal structure and amino acids sequence showed that conserved regions for oligomerization are in the *N*-terminal domain, and in the *C*-terminal domain including two β-helical structures and a disulfide-linked domain. In contrast, it was shown that m2 VacA has a 23 amino-acid insert, which forms an additional β-helix not found in m1 VacA [13]. Genetic analysis of clinical isolates of *H. pylori* strains showed that m1 strains are associated with a higher risk of gastric ulcer than are m2 strains [15]. These different sequences may reflect receptor-binding affinities of m1 and m2 VacA.

VacA induces not only large vacuole formation in the cytoplasmic compartment [16], but also apoptosis, induction of autophagy, cytoskeletal changes, inhibition of antigen presentation, and inhibition of T cell proliferation [3,17,18,19,20,21,22,23,24]. Furthermore, oral administration of VacA to mice have caused gastric inflammation and injury [25]. Previous studies have suggested that the channel-forming activity of VacA is essential for its biological effects [26,27] including autophagosome formation [24,28].

In this review, we show that VacA receptors are associated with the regulation of cellular events including autophagy and apoptosis.

## 2. VacA Receptors on Target Cells

### 2.1. Receptor-Like Protein Tyrosine Phosphatase β (RPTPβ)

Through immunoprecipitation with an anti-VacA antibody, receptor-like protein tyrosine phosphatase (RPTP) β was identified as a VacA receptor [29]. RPTPβ is a membrane protein, which is composed of chondroitin sulfate proteoglycan, with an extracellular region containing a carbonic anhydrase-like domain and a single FNIII domain. In addition, RPTPβ plays an important role in controlling several cellular processes (e.g., cell migration, differentiation, synaptogenesis) [30,31,32]. Although RPTPβ was known to be mainly expressed in the brain, several reports showed that RPTPβ was also expressed in gastric tissue—in particular, the submucosal and muscle layers [33,34]. It has become evident that RPTPβ participates in blood vessel growth and maintenance [35,36,37]. Acid- or alkaline-activation of VacA enhances binding to RPTPβ on the cell surface, resulting in rapid induction of vacuole formation [29]. HL-60 cells are insensitive to VacA [38], but monocytic-like or macrophage-like HL-60 cells, which are generated by treatment with chemical agents (e.g., PMA, VitD3), increase VacA sensitivity by induction of RPTPβ expression on the cell surface. Further, RPTPβ knockdown in macrophage-like HL-60 cells have suppressed VacA activity [39]. The terminal sialic acid modification of RPTPβ plays an essential role in VacA binding [40]. In addition, VacA suppressed phosphatase activity of RPTPβ by increasing the level of Git1 phosphorylation, which causes cell de-attachment [41]. Administration of VacA to wild-type mice resulted in severe gastric damage, including degeneration of the gastric mucosa and acute inflammation, followed by gastric ulcer disease [25]. However, in RPTPβ-knockout mice, tissue damage caused by VacA has not been observed [34]. Thus, these data lead to the hypothesis that Git1 phosphorylation by VacA promotes de-attachment of the gastric epithelial cells and induces gastric damage. These findings suggest that RPTPβ is a functional receptor for VacA that is responsible for gastric disease in *H. pylori* infection.

### 2.2. Receptor-Like Protein Tyrosine Phosphatase α (RPTPα)

In the human kidney tumor cell line G401, a p140-kDa membrane protein (p140) was detected as a VacA-binding protein by immunoprecipitation and identified as RPTPα by amino acid sequence analysis. It has been known that RPTPα is also a transmembrane PTP with a shorter glycosylated extracellular domain than seen with RPTPβ, and a tandem repeat of two cytoplasmic PTP domains [42,43]. Indeed, RPTPα on G401 cells was modified by terminal sialic acid linked to α(2–3)-galactose and galactose-β-(1–3)-*N*-acetyl-galactosamine. As was seen with RPTPβ, neuraminidase treatment attenuated RPTPα binding to VacA. Furthermore, inhibition of RPTPα expression suppressed VacA-induced vacuolating activity [44], suggesting that RPTPα serves as a functional VacA receptor in G401 cells. Recent studies showed that RPTPα interacts with E-cadherin [45], and RPTPα/Src family kinase/Rap1 signaling is involved in recruiting myosin IIB to the zonula adherens and supporting contractile tension [46]. However, the role of RPTPα in VacA-induced disease pathogenesis remains unclear.

### 2.3. Low-Density Lipoprotein Receptor-Related Protein-1 (LRP1)

Recently, we identified LRP1 as a VacA-binding protein by immunoprecipitation with an anti-VacA antibody [28]. LRP1 is a large membrane protein, consisting of extracellular ligand-binding domains (heavy chain region) and a non-covalently associated, light-chain region containing a transmembrane domain and a short cytoplasmic tail. LRP1 is ubiquitously expressed in mammalian tissues [47].

Prior studies demonstrated that VacA induces autophagy in gastric epithelial cells [24,48] and causes apoptosis via mitochondrial damage [49,50,51,52,53]. Interestingly, knockdown of LRP1 by gene silencing attenuated VacA-induced vacuolating activity and inhibited VacA-caused LC3-II generation and PARP cleavage. Knockdown of the other VacA receptors (e.g., RPTPα, RPTPβ, fibronectin) did not inhibit VacA-induced autophagy and apoptosis, indicating that in VacA-treated cells LRP1 mediates autophagy, leading to apoptosis [28]. In addition, the *N*-terminal domain of VacA has anion-channel activity, which is involved in its biological effects such as vacuole formation [21,27,54,55,56]. Anion channel inhibitors suppressed VacA-induced LC3-II generation, indicating that channel activity participated in LRP1-mediated autophagy. In AZ-521 cells, VacA-induced autophagy is Beclin-1-independent [28]. However, cell-signaling pathways that are involved in LRP1-mediated, VacA-induced autophagy remain to be defined.

It has been shown that CagA is directly injected into gastric epithelial cells through a Type IV secretion system and plays an important role in gastric carcinogenesis [57]. CagA is degraded via m1VacA-induced autophagy [58]. Interaction of VacA and LRP1 causes an accumulation of reactive oxygen species (ROS), leading to Akt activation, followed by elimination of p53 by an MDM2-mediated process through the ubiquitin-proteasome pathway, and resulting in formation of autophagic vacuoles. Thus, binding of m1VacA, but not m2 VacA, to LRP1 is an essential step for ROS accumulation and the induction of autophagy. m2 VacA does not bind to LRP1 and thus differs from m1 VacA. Interestingly, in CD44v9 gastric cancer stem-like cells, the amount of intracellular ROS was suppressed by a cysteine transporter (xCT), resulting in inhibition of autophagy and leading to accumulation of intracellular CagA [58]. These findings support the hypothesis that the binding of VacA to LRP1 is responsible for the induction of autophagy as well as the degradation of CagA.

#### 2.3.1. EGFR

Seto and his colleagues reported that VacA-induced vacuolation was inhibited by an anti-EGFR antibody in a dose-dependent manner; cells treated with *H. pylori* culture supernatant were lysed and immunoprecipitated with an anti-EGFR antibody, and VacA fragments (58-kDa and 37-kDa) were detected with an anti-*H. pylori* antibody, which was made using heat-killed whole cells of *H. pylori* as an immunogen [59]. Therefore, it is possible that these anti-*H. pylori* antibodies may detect other factors from *H. pylori* involved in the immunocomplex precipitation by anti-EGFR antibodies. The interaction between EGFR and VacA by immunoprecipitation using anti-VacA needs to be confirmed. VacA interferes with EGF-stimulated increase in actin stress fiber formation and wound re-epithelialization [60], as well as EGFR and ERK1/2 kinase signaling [61]. These findings suggest that VacA may affect EGFR-mediated signaling, leading to inhibition of cell proliferation, renewal of the gastric mucosa, and repair of mucosal injury.

#### 2.3.2. Fibronectin

Fibronectin is a component of the extracellular matrix and involved in multiple cellular processes (e.g., development, tissue repair) [62]. Further, fibronectin is known to play an important role in bacterial invasion and persistent infection [63,64]. Henning *et al.*, demonstrated by ELISA that VacA directly binds to fibronectin [65]. They also showed that VacA in the presence of fibronectin suppressed the adhesion of HeLa cells. The investigators suggested that VacA might affect fibronectin-mediated signal transduction pathways regulated by integrin, FAK, and Src. Indeed, VacA treatment inhibited tyrosine phosphorylation of FAK [66]. However, it is still unknown whether fibronectin directly regulates cell de-adhesion after VacA binding. The extracellular matrix glycosaminoglycans, such as heparin and heparin sulfate, also serve as VacA receptors and participate in cell adhesion [67]. LRP1 is capable of regulating FAK and paxillin, which control cell adhesion and cytoskeleton [68]. Further study will be needed to determine which VacA receptor on target cells regulates focal adhesion turnover.

### 2.4. The Role of Lipid Rafts, Heparan Sulfate, and Glycosaminoglycans in VacA Binding

Previous studies have demonstrated that the interaction of VacA with lipid rafts is a critical step for induction of VacA-induced vacuolating activity [69,70,71,72,73]. Lipid rafts are subdomains of the plasma membrane containing high concentrations of cholesterol and glycosphingolipids [74]. Treatment with methyl-β-cyclodextrin (MβCD), which causes a depletion of cholesterol, leads to suppression of VacA activity [69,70,71,72,73,75]. The interaction between VacA and RPTPβ in AZ-521 cells was not inhibited by MβCD, which suppressed translocation VacA with RPTPβ to lipid rafts [73]. Gupta *et al.*, identified sphingomyelin as a functional VacA receptor, showing that VacA bound directly to sphingomyelin in an ELISA-based assay and that depletion of sphingomyelin by sphingomyelinase decreased VacA-induced vacuolation [76]. By atomic force microscopic analysis, VacA oligomer preferably associated with rafts in dioleoylphosphatidylcholine/sphingomyelin/cholesterol bilayers at low, but not at neutral, pH [69]. Low pH triggers conformational changes in VacA, which reassembles into membrane-spanning hexamers, enabling VacA to form channels across planar lipid bilayers [69,77]. Furthermore, 18-carbon acyl chain variant sphingomyelin-enriched cells are more sensitive than shorter carbon variant sphingomyelin-enriched cells with regard to VacA uptake, intracellular trafficking to a Rab7/Lamp1 compartment, and vacuolating activity [78]. Their proposed model suggests that sphingomyelin in lipid rafts is required for VacA entry and its subsequent transfer to late endosomal/lysosomal compartments.

The anion channel blocker, 5-nitro-2-(3-phenylpropylamino)-benzoic acid (NPPB), did not interfere with translocation of VacA to lipid rafts and activation of p38 MAP kinase/ATF2, but it inhibited VacA uptake and vacuolation. In contrast, phosphatidylinositol-specific phospholipase C (PI-PLC) inhibited several VacA activities including translocation, internalization, activation of p38 MAP kinase/ATF2, and vacuolation. Thus, VacA channel activity in lipid rafts is important for VacA entry and its vacuolating activity [73].

Heparin/heparan sulfate is ubiquitously found at the cell surface and in the extracellular matrix, and participates in various biological functions, such as cell growth and development, angiogenesis, viral infection, and anti-coagulation [79]. Utt and colleagues showed that glycosaminoglycans, such as heparin and heparan sulfate, bound to the *C*-terminal subunit of VacA by surface plasmon resonance (SPR)-based biosensor studies [67]. Their results supported the hypothesis that VacA-induced vacuolation *in vitro* was suppressed in the presence of heparin [80]. Previously, it was shown that RPTPβ interacts with heparin-binding growth factors, such as midkine and pleiotrophin [81], consistent with the possibility that RPTPβ is modified by heparin/heparin sulfate-like motif and is involved in VacA binding. These findings indicate that heparin/heparan sulfate plays a role in VacA uptake by cells.

Other groups have shown that glycosylphosphatidylinositol-anchored proteins (GPI-APs) play an important role in VacA activity [72,75]. Treatment of cells with phosphatidylinositol-specific phospholipase C (PI-PLC) to remove GPI-APs did not affect VacA binding to the cell surface, but impaired VacA activity [72]. Thus, GPI-APs do not directly associate with VacA. However, they are involved in toxin entry into cells by endocytosis.

### 2.5. CD18 on T Lymphocytes

VacA has immunosuppressive effects, including inhibition of antigen presentation [82] and suppression of T and B lymphocyte proliferation [83]. VacA impairs nuclear translocation of nuclear factor of activated T cells (NFAT), leading to inhibition of interleukin-2 (IL-2) transcription and its signaling pathway [84,85,86].

Sewald *et al.*, reported that, in immunoprecipitation experiments, a β2 integrin (CD18) subunit interacts with VacA and co-localizes with VacA by confocal microscopic analysis, suggesting that CD18 is a functional receptor for VacA in T cells and is required for VacA internalization [87]. They proposed a mechanism for VacA endocytosis into T cells [88]. After binding to a cell surface factor (e.g., sphingomyelin, GPI-APs), VacA interacts with CD18 and then moves to lipid rafts. In this domain, phosphorylation of the cytoplasmic region of CD18 by PKCη or PKCξ triggers VacA endocytosis via activation of small GTPases Cdc42 and Rac1, resulting in its translocation into cells through a clathrin-independent pathway [88]. These findings were supported by the observation that VacA bound to αMβ2 integrin by ELISA [89]. It was observed that the VacA intermediate region in the *N*-terminal p33 domain, which is designated type i1 and i2 [90], plays a crucial role in VacA’s binding to Jurkat T cells. Although i1 VacA bound with significantly higher affinity to Jurkat T cells than did i2 VacA, these isoforms bound to αMβ2 integrin without significant differences. These results suggest that the interaction between VacA and target cells involves not only the *C*-terminal p55 domain but also the intermediate region; the i-region may regulate VacA recognition of alternative receptors [89].

In addition, VacA has been shown to inhibit (a) activation-induced proliferation of CD4^+^ T cells, CD8^+^ T cells, and B cells [83]; (b) maturation of lipopolysaccharide (LPS)-treated dendritic cells through a restoration of E2F1 signaling [91]; and (c) antigen presentation by antigen-presenting cells [82]. 

### 2.6. Multimerin 1 on Platelets

Although it is well known that *H. pylori* infection is associated with immune thrombocytopenia purpura, the detailed mechanism remains unknown. Previous studies have shown that the expression of CD62P increases on platelets in *H. pylori*-infected mice and humans [92] and that it is decreased by eradication of *H. pylori* [93]. CD62P is a surface marker of activated platelets and activated endothelium [94]. Thus, *H. pylori* infection induces platelets activation. Satoh *et al.*, reported that VacA, but not heat-inactivated VacA, induced CD62P expression on platelets and, by immunoprecipitation, identified a new VacA-binding protein, multimetrin 1, on platelets as a candidate for platelet activation [95]. Although human platelet membranes expressed both RPTPβ and CD18, they did not detect binding between VacA and these potential receptors. It is possible that the sugar modification of these proteins on platelets is different from that found in other tissues. The large number of enzymatic steps involved in glycosylation and their activities vary by cell type and intracellular compartment, resulting in the synthesis of glycoproteins with a variety of glycan structures [96]. Indeed, thirteen glycosylation sites were found on multimerin 1 in human platelets [97]. Sugar modification plays an important role in the interaction between VacA and its receptors [40]. The terminal sialic acids especially play a pivotal role in VacA binding.

Multimerin1 is a homopolymeric adhesive protein that is found in platelets and endothelial cells. After platelets are activated, multimerin 1 is released from the platelets and binds to the activated platelets, which enhances their adhesion at sites of vascular injury [98,99]. However, the mechanism by which VacA induces platelet activation remains unknown.

## 3. Conclusions

**Figure 1:**
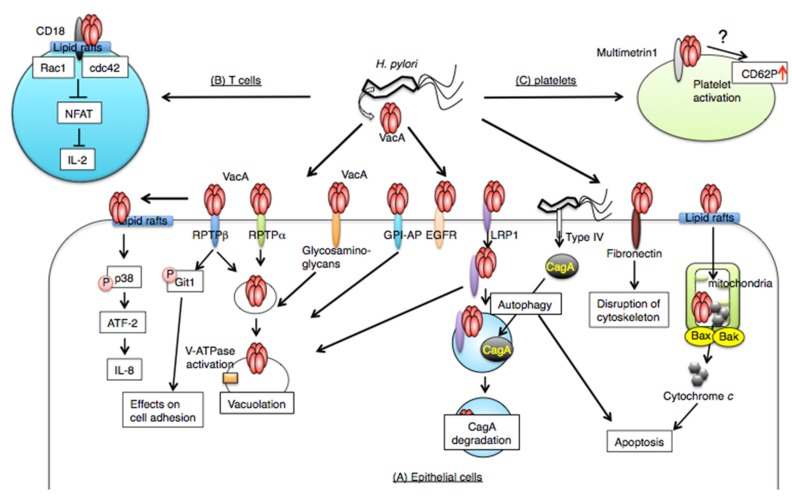
VacA receptors. After VacA binding to receptors, it is translocated into cells by endocytosis and is responsible for multiple effects. (**A**) In epithelial cells, VacA binds to several receptors on target cells. Most of the receptors (e.g., RPTPα, RPTPβ, glycosaminoglycan, GPI-AP, EGFR, LRP1, fibronectin, sphingomyelin, lipid rafts) are involved in VacA uptake and vacuolating activity. In addition, lipid rafts are associated with VacA-induced apoptosis, which is also controlled by LRP1. VacA-induced apoptosis is caused by Bax/Bak conformational changes, leading to cytochrome *c* release. Interaction between VacA and LRP1 induces autophagy, which regulates stability of CagA released into cells by the *H. pylori* type IV secretion system. Signaling of VacA bound to fibronectin regulates cell adhesion and cytoskeletal organization. (**B**) On T cells, a β2 integrin (CD18) subunit interacts with VacA, leading to activation of cdc42 and Rac1, followed by VacA uptake. Intracellular, VacA impairs NFAT, leading to inhibition of IL-2 transcription. (**C**) Multimetrin 1 is a candidate for VacA receptors on platelets. Effects on VacA–multimetrin 1 interactions may be facilitated by CD62P expression on platelets.

In this review, we show that *H. pylori* VacA recognizes tissue-specific, cell surface receptors to gain entry into target cells, leading to a perturbation of various cell biological processes. The relationships between VacA and its receptors are summarized in Figure 1. Some receptors participate in vacuole formation, and other receptors are involved in mitochondrial dysregulation or functional disruption of cell signaling. These receptors are differentially expressed in tissues. Therefore, we need to investigate the structural and functional changes of the receptors caused by VacA and their effects on the biological processes. Thus, understanding the mechanisms will provide important information on the association between toxicity and VacA receptors.

It seems that VacA interacts with not only the core protein region of the receptors but also their glycosylation motifs. Many bacterial toxins use sugar moieties as receptors [100]. By investigating the kinds of sugar structures that have a high affinity for VacA, we might be better able to design compounds to inhibit VacA-induced damage.
